# Heparin-induced thrombocytopenia and acute colonic pseudo-obstruction after therapeutic anticoagulation in a very elderly patient with hip fracture: a case report

**DOI:** 10.1186/s12891-020-3117-8

**Published:** 2020-02-07

**Authors:** Yijun Liu, Hao Lu, Hailin Xu, Zhongguo Fu, Dianying Zhang, Baoguo Jiang

**Affiliations:** Department of Orthopaedics and Trauma, Peking University People’s Hospital, Beijing, 100044 China

**Keywords:** Hip fracture, Low molecular weight heparin, Heparin-induced thrombocytopenia, Digestive hemorrhage, Acute colonic pseudo-obstruction, Case report

## Abstract

**Background:**

Hip fractures have become a severe public health problem, especially in very elderly patients. Most of them are treated with low molecular weight heparin as prophylaxis or treatment of venous thromboembolism. Heparin-induced thrombocytopenia is one of the complications induced by low molecular weight heparin, which may cause poor prognosis. However, there is not enough awareness for heparin-induced thrombocytopenia in very elderly trauma patients.

**Case presentation:**

We report a case of hip fracture with heparin-induced thrombocytopenia in a very elderly patient. The patient developed heparin-induced thrombocytopenia, digestive hemorrhage and acute colonic pseudo-obstruction after the use of low molecular weight heparin, which eventually led to death.

**Conclusions:**

This is the first case report of digestive hemorrhage and acute colonic pseudo-obstruction in heparin-induced thrombocytopenia patients with major trauma. This case highlights the severity of HIT in very elderly patients with hip fractures using low molecular weight heparin, and the need for platelet monitoring in these patients. We indicate that there may be a correlation of pathogenesis between digestive hemorrhage and acute colonic pseudo-obstruction in heparin-induced thrombocytopenia patients.

## Background

As the number of elderly increases, hip fractures become a severe public health problem, especially in very elderly patients [1]. The preoperative incidence of venous thromboembolism in hip fracture patients is approximately 18.4–19.5% [2, 3]. Several current guidelines recommend low molecular weight heparin (LMWH) as an optimal form of venous thromboembolism (VTE) prophylaxis or treatment in patients with hip fractures [4–6]. Very elderly (age > 80 years) trauma patients have worse general conditions and higher risks of heparin related complications, which may lead to poor prognosis [7–9]. However, HIT in very elderly trauma patients does not receive enough attention. We report a case of hip fracture in a very elderly patient who developed serious complications, such as HIT, digestive hemorrhage and acute colonic pseudo-obstruction (ACPO) after the use of LMWH. We obtained consent for publication from the patient's son.

## Case presentation

**Fig. 1:**
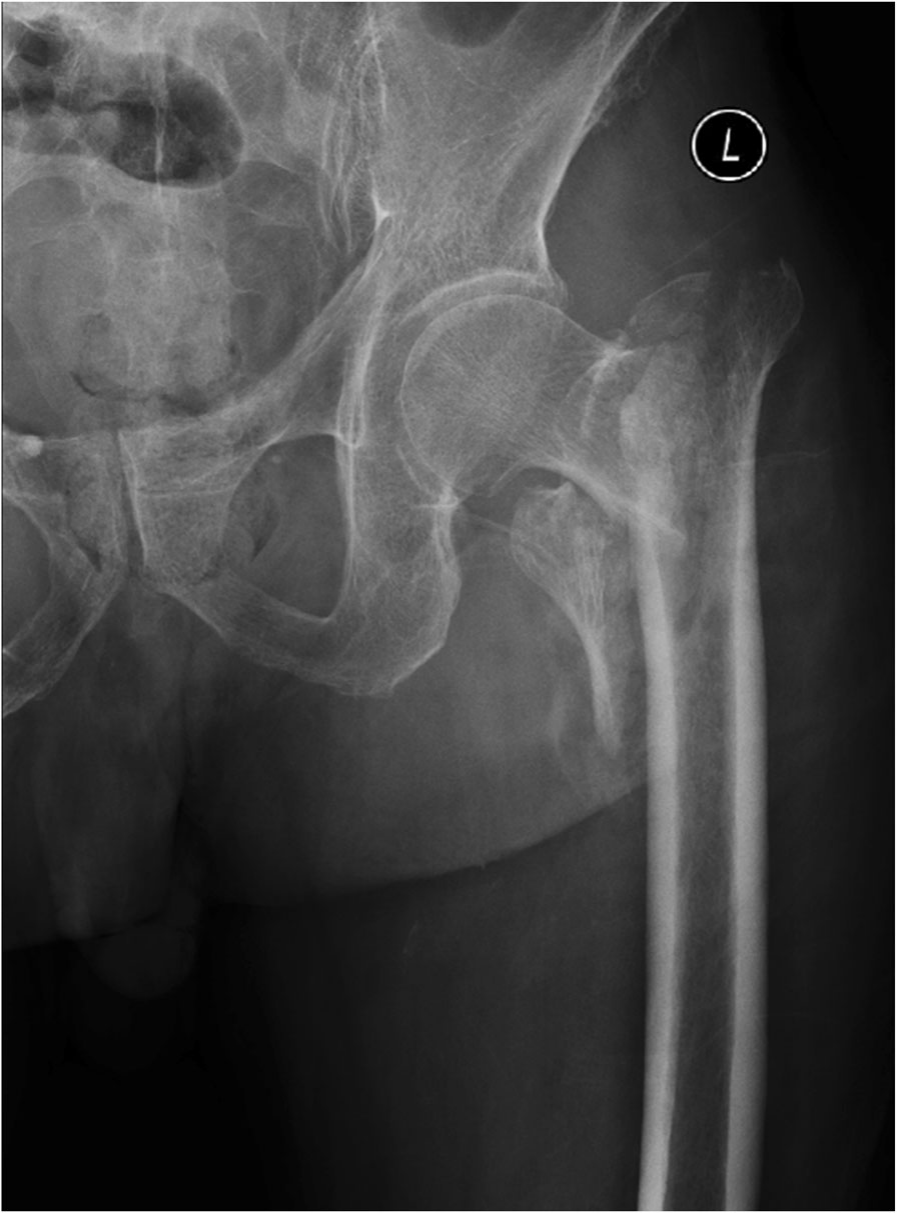
X-ray indicated left intertrochanteric fracture

**Fig. 2:**
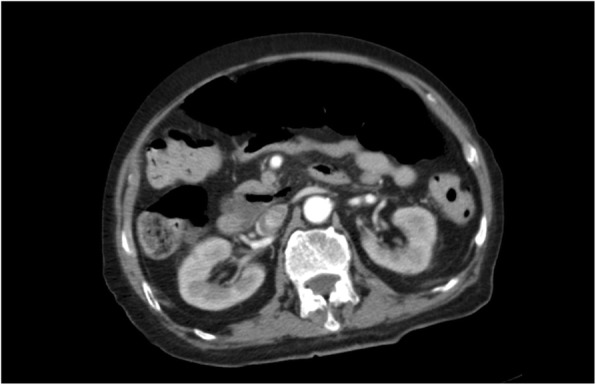
Abdominal CT scan showed colonic dilatation with no sign of thromboses and mechanical obstruction

An 84-year-old male patient fell while walking and suffered left intertrochanteric fracture (Fig. 1). He refused the surgery recommendation, chose to remain bedridden. Physical therapy for prophylaxis of thromboembolism at home was prescribed. Ten days later, his left calf swelled, and venous thrombosis was identified by ultrasound in popliteal vein and posterior tibial veins. The patient was admitted to our department 13 days after the injury to evaluate and improve medical fitness and prepare for internal fixation. The patient had a medical history of cerebral infarction more than 10 years ago, and long-term use of aspirin. The platelet count was 349 × 10^9/L, and the haemoglobin count was 112 g/L on the first day of admission (Table 1). Aspirin was stopped and LMWH (FRAGMIN, Pfizer) 5000 IU was given twice daily as therapeutic anticoagulation therapy. Moreover, the inferior vena cava filter was placed. Unfortunately, serious blood shortage happened which led to the postponement of the internal fixation. The patient had abdominal distention and melena on the 16th day after admission (Table 1). He developed hematochezia 3 h later without peritoneal irritation. Redness and swelling were found at the LMWH injection site. The platelet count was 3 × 10^9/L, and the haemoglobin count was 98 g/L. The sum of the 4 T’s scores was 6. Autoantibodies, anti-ds DNA antibody, and other tests for differential diagnosis were normal. Therefore, we made the clinical diagnosis of HIT, digestive hemorrhage, VTE, and intertrochanteric fracture. We stopped LMWH therapy and underwent gamma globulin infusion (0.4 g/kg, iv), methylprednisolone infusion (60 mg, iv, QD), platelet transfusion and total parenteral nutrition (TPN). After that, the platelet count increased steadily, and the digestive haemorrhage gradually stopped. On the 24th day after admission (Table 1) (the 5th day of the use of gamma globulin) the platelet count recovered to 60 × 10^9/L, and the haemoglobin count recovered to 96 g/L. On the 35th day after admission (Table 1), the patient developed abdominal distending pain. Physical examination indicated the weakening of bowel sounds without abdominal tenderness. The platelet count was 87 × 10^9/L. The haemoglobin count was 108 g/L. The WBC count was 12.8 × 10^9^/L, and the potassium concentration was 5.59 mmol/L. Abdominal X-ray showed colonic dilatation. The abdominal CT scan showed colonic dilatation with no sign of thromboses and mechanical obstruction, such as thickening of the colonic wall or thrombosis of mesenteric vessels (Fig. 2). Therefore, we performed a multidisciplinary consultation and made a diagnosis of ACPO and performed conservative treatments, such as TPN and gastrointestinal decompression therapy. On the 39th day after admission (Table 1), the patient developed high fever and unconsciousness without acute abdominal pain and tenderness. The platelet count was 14 × 10^9/L. The haemoglobin count was 95 g/L, and the WBC count was 4.8 × 10^9^/L. The serum lactate was 4.0 mmol/l, the blood pressure was 90/63 mmHg, and heart rate was 98 beats per minute, which suggested of septic shock. We diagnosed the patient with gut-origin septic shock and micro-angiopathic hemolysis. The conservative treatments, such as gastrointestinal decompression, TPN, anti-infection and somatostatin were ineffective, and the patient died 4 days later.

**Table 1:** Summary of the laboratory results

Days after admission	1	16	24	35	39
Platelet count(10^9/L)	349	3	60	87	14
Haemoglobin count(g/L)	112	98	96	108	95
WBC (10^9/L)	10.3	7.6	9.3	12.8	4.8
K^+^ (mmol/L)	4.1			5.59

## Discussion and conclusions

HIT is a clinical complication after exposure to heparin for approximately 5–14 days, which results from antibodies directed against antigenic complexes of platelet factor 4 and heparin [10]. Patients exposed to LMWH seldom develop HIT with an incidence rate of approximately 0.2–0.54% [11, 12]. The platelet count is rarely less than 15,000/μL [13]. Only approximately 2.7% of HIT patients develop digestive hemorrhage [14]. Elderly patients with hip fracture or surgery rarely develop ACPO with an estimated prevalence of 0.067–0.29% [15–17] indicating that this case is very rare, and these complications might not be independent. This is the first known case report about the combination of digestive hemorrhage and ACPO in HIT patients.

This case indicates that very elderly trauma patients may have a worse prognosis. As literature documented, comorbidities might contribute to the development or exacerbation of HIT. Sun X et al. [18] found that HIT is more likely to happen in patients with clinical heart failure and increased body mass index. Joseph et al. [19] found that acutely ill medical patients are at higher risk of HIT, and HIT-related major bleeding risk will increase in patients in critical care units and dialysis dependent.

It remains controversial whether platelet count monitoring is necessary on isolated HIT patients. In the guidelines of the American College of Chest Physicians (ACCP) [20], protocols of platelet count monitoring in patients using heparin are based on the incidence of HIT. The patient population is divided into the postoperative group and medical patients group according to the incidence of HIT. This classification excludes preoperative elderly patients with major trauma, such as hip fractures. However, these patients usually have more comorbidities and worse surgical tolerance [8]. Therefore, it takes longer to evaluate and improve medical fitness for the procedure. Moreover, according to the American Society of Hematology 2018 guidelines [21], patients receiving LWMH after major trauma belong to intermediate-risk group, and platelet count monitoring every 2 to 3 days was recommended. However, it is a conditional recommendation with very low certainty evidence. This case indicates that very elderly trauma patients may have a worse prognosis of HIT than younger patients. Therefore, a routine analysis platelet count may be necessary for these patients.

It is still controversial whether additional non-heparin anticoagulant should be used in patients with isolated HIT and digestive bleeding after discontinuation of heparin. The most common complication of HIT is thrombosis, with an incidence of more than 50% [20, 22]. Therefore, it is difficult to strike the risk-benefit balance of bleeding control and thrombosis prophylaxis. The 9th American College of Chest Physicians evidence-based clinical practice guideline recommends the use of lepirudin or argatroban or danaparoid over the discontinuation of heparin. However, it remains uncertain whether non-heparin anticoagulant will increase the risk of major bleeding [20]. The American Society of Hematology guideline panel recommends reducing the non-heparin anticoagulant intensity from therapeutic to prophylactic if the patient is at high risk of bleeding [21]. We did not initiate non-heparin anticoagulant because of severe digestive hemorrhage. We hypothesise that the absence of non-heparin anticoagulant might lead to a risk of micro-thrombosis and hypoperfusion of the bowel wall, which might led to ACPO. Therefore, we suggest further study to clarify the relationship between the discontinuation of heparin and ACPO.

ACPO is a rare clinical syndrome characterised by acute colonic dilatation without mechanical obstruction [15, 23]. The imbalance of the autonomic nervous system nervous is the universal pathogenesis theory of ACPO. Other studies suggest that ACPO might be related to hypoperfusion, abnormal hormone levels and decreases in pacemaker cells [15, 23]. This patient had a platelet transfusion history and did not have an additional non-heparin anticoagulant after discontinuation of heparin, which led to a higher risk of thrombosis. We hypothesise that hypoperfusion caused by micro-thrombosis and a decrease in pacemaker cells caused by digestive haemorrhage may play a role in the development of ACPO. However, trauma and being bedridden long-term may also lead to the imbalance of the autonomic nervous system [17]. Therefore, HIT and ACPO, in this case, might be two independent complications. In any case, we suggest future studies to focus more on links between ACPO and HIT trauma patients.

In conclusion, this is the first case report of digestive hemorrhage and acute colonic pseudo-obstruction in heparin-induced thrombocytopenia patients with major trauma. This case suggests that HIT in very elderly trauma patients might cause severe complications, such as gastrointestinal bleeding and ACPO, which would lead to poor prognosis. Therefore, we suggest a routine analysis of plate count in the very elderly patients with major trauma, and an interdisciplinary approach might be needed in the case of HIT. Moreover, there may be correlations in pathogenesis between digestive hemorrhage and ACPO in trauma patients with HIT.

## Data Availability

The datasets in this case report are not publicly available due to the protection of patient’s information. However, the reasonable requests will be met by the corresponding author.

## Abbreviations

ACPO: Acute colonic pseudo-obstruction
HIT: Heparin-induced thrombocytopenia
LMWH: Low molecular weight heparin
TPN: Total parenteral nutrition
VTE: Venous thromboembolism
